# Observations on the Poisonous Vegetables Which Are Either Indigenous in Great Britain, or Cultivated for Ornament

**Published:** 1781-05

**Authors:** 


IV. *Obfervations on the poifonous Vegetables which are either indigenous in Great Britain, or cultivated for Ornament. By* B. Wilmer, *Surgeon.* 8vo. Longman, *London.* 103 pages, 2s.

FORTUNATELY, the number of poifonous herbs is very fmall. Even of thefe the dreadful effects may be prevented, by an imimmediate and proper attention, or removed, though they have partly taken place. They appear to act by an impreſſion on the nervous ſyſtem, rather than by an inflammation of the ſtomach and duodenum as mineral poiſons do, which from this beginning produce thoſe other intervening ſymptoms that uſually end in death. In different conſtitutions, however, they will have various, and ſometimes oppoſite effects.

The vegetable poiſons deſcribed in the work before us, are arranged in two diviſions. The firſt includes thoſe from which maniacal ſymptoms may be expected, or the various nervous affections, from a vertigo to a fatal apoplexy. The plants of this claſs are, the *hyoſcyamus niger*, *belladonna*, *napellus cæruleus*, *cynocrambe*, *ſtramonium*, *cicuta major*, *agaricus muſcarius*, *fungus piperatus*. The other claſs conſiſts of ſuch as produce epileptic ſymptoms. A loſs of underſtanding, of ſpeech, and of all the ſenſes, commonly taking place in a few minutes after theſe poiſons are in the ſtomach, and death cloſing the ſcene in the ſhort period of one hour or two. The plants of this claſs known in Great Britain, and enumerated by our author, are the *oenanthe crocata*, *cicuta aquatica*, and *lauro-ceraſus*. The two firſt only are natives of our our iſland; the laſt is moſt fatal, and requires a chemical preparation. Its poiſon may therefore in ſome meaſure be ſaid to be an invention of art.

To his account of each poiſonous herb, Mr. Wilmer has prefixed the ſynonyma of different botanic authors; and after giving a deſcription of the plant, adds ſuch remarks on its effects as his own experience, or the works of preceding writers, have ſuggeſted to him.

In treating of the common henbane, he obſerves, that it is frequently found upon dunghills, and that its roots mixt with manure are introduced into our gardens, where he ſuppoſes them to have been often miſtaken for thoſe of parſnip, which they much reſemble. His friend Mr. Harrold once ſaw two women, who from eating the ſuppoſed roots of parſnip became maniacal, and were ſo furious, that ſtrict confinement was neceſſary for ſeveral days.

In the year 1765, the family of a farmer at Loughton in Buckinghamſhire, conſiſting of ſix perſons, dined upon pudding, boiled meat, and the roots of parſnip. Soon after dinner they all became ill, and in two hours our author was a witneſs of the following ſcene.—Mrs. York (the farmer's wife) was upon a bed with all the

ſymptoms

ſymptoms of apoplexy. Two of the children were ſtupid, and appeared like thoſe intoxicated with ſpirituous liquors. A man-ſervant and the maid, with uncommon agitation of mind, were dancing about the room with all the appearance of maniacal perſons. A middle aged man (the ſhepherd) had dined with the reſt, and after dinner went about his buſineſs in the fields, where he was found ſtaggering like a man intoxicated.

Mr. Wilmer attempted to give an emetic to the man-ſervant, but he had no ſooner received it into his mouth than he ſpit it out again. Five grains of emetic tartar, diſſolved in water, were then conveyed into the ſtomach by means of a funnel, and he ſoon vomited up large quantities of the roots. In a ſhort time he recovered the uſe of his reaſon, and complained of nothing more than a ſlight head-ach. An emetic was given to all the reſt, except Mrs. York, and after the ſtomach had ejected its contents, they recovered in a very ſhort ſpace of time.

Mrs. York had never eat any parſnips before in her life; but being prevailed upon, unfortunately, to taſte them, ſhe took more than any one of the family. All attempts to convey medicine into her ſtomach were ineffectual. Acrimonious and purgative glyſters were injected, without producing any evacuation. The moſt powerful ſtimulants were applied to various parts of the body, without exciting any apparent effect. In the evening the apoplectic ſnoring increaſed, attended with a quick pulſe; her extremities were warm and moiſt with ſweat. During the night, the difficulty of reſpiration was accompanied with a rattling in the bronchiæ; the noſtrils were compreſſed, her feet became cold, and at ſix o'clock in the morning ſhe died. Our author could not obtain leave to open the body.

Suſpecting that the roots of ſome poiſonous plant had been mixed with the parſnips, he deſired to ſee ſome of them; and upon an accurate examination perceived them evidently of two kinds. As the roots at that time were not furniſhed with leaves, he took them home and planted them in his garden. Some of them proved to be the *paſtinacha ſativa*, or garden parſnip, and the other the *hyoſcyamus niger*, or common henbane.

In his account of the *napellus cœruleus*, or blue monkſhood, the extract of which has been ſo much extolled by Dr. Storck and Dr. Collin as a cure a cure for rheumatiſm, our author informs us, that he gave it a fair trial in two caſes of chronic rheumatiſm, and in the doſes recommended by Dr. Storck, but without experiencing any good or ill effect from it.

When any of the narcotic vegetable poiſons have been unfortunately taken, our author obſerves that the indications of cure will be, 1. To unload the ſtomach by the ſpeedy adminiſtration of an active emetic. 2. To procure ſtools, either by proper cathartics, if the patient can ſwallow them, or by the injection of ſtimulating clyſters. 3. To correct and counteract the ſedative effects of the poiſon, by giving from time to time draughts of ſome vegetable liquor, weak ſparkling cyder or perry. And, 4. If any paralytic ſymptoms ſhould remain, or the muſcular action be much impaired, proper ſtimuli ſhould be applied, ſuch as ſinapiſms and bliſters, but more particularly the uſe of electricity is indicated.

By obſerving theſe rules, he once ſaw a patient who had taken two ounces of the thebaic tincture, perfectly recover in two days.

The poiſons of the firſt claſs, in general, have a viroſe diſagreeable ſmell and taſte; on the contrary, thoſe of the ſecond would appear by the evidence evidence of the ſenſes to be perfectly harmleſs. But they ſpeedily occaſion epileptic ſymptoms. Of all epilepſies, remarks our author, theſe are the moſt fatal; of all poiſons, theſe are the moſt deadly. Pleaſant to the taſte, or inoffenſive to the palate, they paſs unſuſpected into the ſtomach; as ſoon as they take poſſeſſion there, the two orifices are ſhut up by ſpaſms; nothing can be expelled, nor can any thing be got in: all poſſibility of relief is cut off; and ſhould that principle inherent in animal life, which tends to throw off every thing injurious to the machine, act, it produces thoſe ineffectual heavings and ſtruggles, which anſwer no other purpoſes than to accelerate and increaſe the effects of the poiſon.

At a time when putrefaction is far advanced, and at a diſtant period from death, ſhould the face be diſcovered of an intenſe black colour, it may naturally be aſked, from whence it ariſes. Does putrefaction occaſion it? To this queſtion the author replies, that the blackneſs of the face is occaſioned by putrefaction, but not by that only. He obſerves, that if convulſions precede death, and the body becomes very putrid after it, the effect may be produced, as it depends on an interruption of the circulation through the lungs lungs by an epileptic ſpaſm. When this happens, the left ventricle of the heart will exert a power to overcome the reſiſtance; but as no more blood can be received by the vena cava, already overcharged, it muſt neceſſarily either be propelled into veſſels which in a healthy ſtate refuſe admiſſion to red blood, or the ſmall arteries terminating in red veins will be ruptured, and their contents conſequently thrown out into the cellular membrane under the ſkin. At the time, or ſoon after death, the extravaſated blood is not viſible through the ſkin: but when the proceſs of putrefaction takes place, an inteſtine commotion enſues; an elaſtic air diſtends the body; the ſtagnant blood is rendered both thinner and blacker; it ſoaks through the cutis, is refuſed a paſſage by the minute pores of the cuticle; and ſpreading abroad, dyes the rete mucoſum of a black colour.

In his account of the *lauro-ceraſus*, Mr. Wilmer relates three experiments made by himſelf on different animals with laurel water. In the firſt experiment, two ounces of it were given to a large ſtrong dog. Two minutes after taking it the animal was convulſed; but after violent efforts to vomit brought up the greateſt part of it, and in the ſpace of three or four minutes recovered.

covered. One ounce more of the water was then given him, with which he was fooner and more affected than with the firft dofe. He breathed with difficulty, vomited, and his head was drawn backward. He fell down and was generally convulfed; but in lefs than half an hour perfectly recovered. The fecond experiment was on a young greyhound. One ounce of the water was poured into the dog's throat, and as foon as it was fwallowed the animal fell down upon its fide, and without the leaft perceptible motion was dead in a moment. The fubject of the third experiment was a mare, aged 28 years. Within a minute from the time of her fwallowing a pint and a quarter of laurel water, fhe feemed affected. In two minutes fhe fell down, and fhortly after was violently convulfed. The convulfions continued about five minutes, at the end of which time fhe lay ftill, but her breathing was very quick and laborious, and her eyes were affected with continual fpafms. Four ounces more of the water were then given to her, and in about fifteen minutes from the time of her firft feizure, fhe expired. After death, the lungs were found remarkably full of blood, the fmall veffels upon their furface being as viſible as if they had been injected with wax.

Although the poiſon of laurel appears to conſiſt in the eſſential oil brought over by diſtillation, yet it is much to be ſuſpected that an infuſion of its leaves may in ſome caſes, and ſome conſtitutions, prove injurious. They have been in common uſe to give a flavour to cuſtards, &c. but from an inſtance of their effects related by our author, this practice ſhould not be continued. He had occaſion it ſeems to ſee a young lady of an irritable habit, who was affected in the night with ſickneſs, ſucceeded by cold ſweats, an irregular pulſe, and ſuch other ſymptoms as led him to ſuſpect that ſhe had taken ſomething extremely noxious into her ſtomach. It appeared, that ſhe had eaten nothing the preceding evening but cuſtard; but, upon examination, Mr. Wilmer found that the cuſtards were very ſtrongly flavoured with laurel leaves. She continued ill a few days, and afterwards perfectly recovered.

